# L1 drives HSC aging and affects prognosis of chronic myelomonocytic leukemia

**DOI:** 10.1038/s41392-020-00279-4

**Published:** 2020-09-19

**Authors:** Ying Wang, Jin-ping Zheng, Ying Luo, Junyi Wang, Lingjie Xu, Jinyong Wang, John M. Sedivy, Zhangfa Song, Hu Wang, Zhenyu Ju

**Affiliations:** 1 Key Laboratory of Aging and Cancer Biology of Zhejiang Province, Institute of Aging Research, School of Medicine, Hangzhou Normal University, Hangzhou, 311121 China; 2 Department of Public Health and Preventive Medicine, Changzhi Medical College, Changzhi, Shanxi 046000 P. R. China; 3 Key Laboratory of Regenerative Medicine of Ministry of Education, Guangzhou Regenerative Medicine and Health Guangdong Laboratory, Institute of Aging and Regenerative Medicine, Jinan University, Guangzhou, 510632 China; 4 CAS Key Laboratory of Regenerative Biology and Guangdong Provincial Key Laboratory of Stem Cell and Regenerative Medicine, Guangzhou Institutes of Biomedicine and Health, Chinese Academy of Sciences, Guangzhou, China; 5 Department of Molecular Biology, Cell Biology and Biochemistry, Brown University, Providence, RI 02903 USA; 6 Department of Colorectal Surgery, Sir Run Run Shaw Hospital, Zhejiang University, Hangzhou, China

**Keywords:** Ageing, Haematological cancer

**Dear Editor,**

Telomere attrition is one of the hallmark of aging. Late-generation *Terc* knockout mice exhibit impaired hematopoiesis,^1^ while the underling mechanisms remain poorly understood. Retrotransposon long interspersed element-1 (L1) is the only human retrotransposable elements capable of autonomous retrotransposition, and evolutionarily inactive. Recent studies reported that L1 is derepressed during the aging process with redistribution and reorganization of the heterochromatin.^2^ Considering that telomere shortening can cause chromosome instability and rearrangements,^3^ we speculate that L1 may play a role in impaired hematopoiesis in telomere dysfunctional mice.

As expected, we found that the expression of L1 were significantly increased in bone marrow (BM) cells from the 3rd generation telomerase deficient (G3*Terc*^−*/*−^) mice compared with that of wild type (WT) mice (Fig. 1a, Supplementary Fig. S1a), which was associated with the decreased CpG methylation at L1 promoter region in G3*Terc*^−*/*−^ mice (Supplementary Fig. S1b). Additionally, we observed that L1 was accumulated in cytosol of BM cells in G3*Terc*^−*/*−^ mice (Fig. 1b, Supplementary Fig. S2a). To determine whether L1 activates cGAS signaling in mice with telomere dysfunction, we examined 2’3’-cGAMP expression and the phosphorylation levels of TBK1, IRF3, and NF-κB p65, the canonical 2’3’-cGAMP downstream, and found that cGAS signaling was significantly activated(Fig. 1c, Supplementary Fig. S2b). Consequently, the expression of type I interferon IFNα and IFNβ, downstream interferon-stimulated genes (CXCL1 and CXCL10), and other cytokines (IL-6, IL-17A, and TNFα) were dramatically upregulated in BM cells and plasma in G3*Terc*^*−/−*^ mice (Fig. 1d, Supplementary Fig. S2c, d). Furthermore, we generated G3*Terc*^−/−^*cGAS*^−/−^ double knockout mice, and found the reduced type I interferon response and decreased expression of cytokines in G3*Terc*^−/−^*cGAS*^−/−^ mice (Fig. 1e, Supplementary Fig. S2e, f). These results demonstrate that L1-cGAS signaling is responsible for telomere dysfunction induced inflammation.

**Fig. 1:**
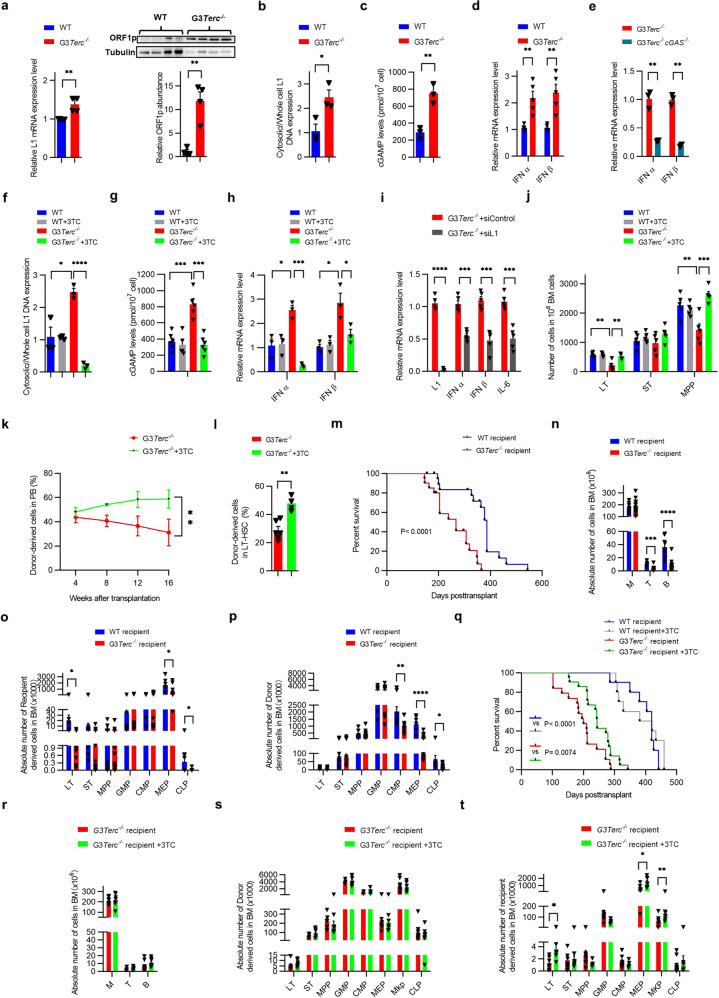
L1 drives HSC aging and affects prognosis of chronic myelomonocytic leukemia. **a** Quantitative real-time PCR (Q-PCR) analysis of the relative mRNA expression level of L1 in BM of WT and G3*Terc*^*−/−*^ mice (*n* = 4, left). The protein levels of L1 (ORF1p) was determined by western blot in BM of WT and G3*Terc*^−*/−*^ mice. The graph represents the relative ORF1p protein abundance (right). **b** Q-PCR analysis of the relative cDNA levels of L1 in BM cytoplasmic fraction of WT and G3*Terc*^−*/−*^ mice (*n* = 3). **c** 2’3’-cGAMP levels were determined by LC-MS/MS in BM of WT and G3*Terc*^−*/−*^ mice (*n* = 3). **d** Q-PCR analysis of the relative mRNA expression levels of IFNα and IFNβ in WT and G3*Terc*^−*/−*^ mice (*n* = 5). **e** Q-PCR analysis of the relative mRNA expression levels of IFNα and IFNβ in G3*Terc*^−*/−*^ and G3*Terc*^*−/−*^c*GAS*^*−/−*^ mice (*n* = 3). **f** Q−PCR analysis of the relative cDNA levels of L1 in BM cytoplasmic fraction of WT or G3*Terc*^−*/−*^ mice treated with or without 3TC (*n* = 3). **g** 2’3’-cGAMP levels were determined by LC-MS/MS in BM of WT or G3*Terc*^*−/−*^ mice treated with or without 3TC (*n* ≥ 5). **h** Q-PCR analysis of the relative mRNA expression levels of IFNα and IFNβ in BM of WT or G3*Terc*^*−/−*^ mice treated with or without 3TC (*n* = 3). **i** Q-PCR analysis of relative mRNA expression levels of L1, IFNα, IFNβ, and IL-6 in control and L1 depleted BM cells of G3*Terc*^*−/−*^ mice (*n* = 5). **j** Numbers of LT (CD34^−^Flt3^−^LSK), ST(CD34^+^Flt3^−^ LSK) and MPP (CD34^+^Flt3^+^ LSK) per million BM cells of WT, or G3*Terc*^*−/−*^ mice treated with or without 3TC (*n* = 5). **k** Percentage of donor-derived PB cells of G3*Terc*^*−/−*^ mice treated with or without 3TC at the indicated time points in competitive transplantation assay (*n* ≥ 3). **l** Percentage of donor-derived LT in BM of G3*Terc*^*−/−*^ mice and 3TC-treated G3*Terc*^*−/−*^ mice 16 weeks after transplantation (*n* ≥ 4). **m** Survival curve of WT recipient mice and G3*Terc*^*−/−*^ recipient mice following transplantation (*n* = 20). **n** Absolute number of M, T, and B cells in BM of WT recipient mice and G3*Terc*^*−/−*^ recipient mice (*n* ≥ 8). **o** Absolute number of LT, ST, MPP, GMP (granulocyte/monocyte progenitor CD34^+^CD16/32^+^Sca1^−^c-Kit^+^ Lin^−^), CMP (common myeloid progenitor, CD34^+^CD16/32^−^Sca1^−^c-Kit^+^Lin^−^), MEP (megakaryocyte /erythrocyte progenitor, CD34^−^CD16/32^−^Sca1^−^c-Kit^+^Lin^−^), and CLP (common lymphoid progenitor, Flt3^+^ IL-7R^+^c-Kit^mid^Sca1^mid^Lin^−^) in recipient-derived BM of WT recipient mice and G3*Terc*^*−/−*^ recipient mice (*n* ≥ 7). **p** Absolute number of LT, ST, MPP, GMP, CMP, MEP, and CLP in donor-derived BM of WT recipient mice and G3*Terc*^*−/−*^ recipient mice (*n* ≥ 5). **q** Survival curve of WT recipient or G3*Terc*^*−/−*^ recipient mice treated with or without 3TC following transplantation (*n* ≥ 10). **r** Absolute number of M, T, and B cells in BM of G3*Terc*^*−/−*^ recipient mice treated with or without 3TC (*n* ≥ 7). **s** Absolute number of LT, ST, MPP, GMP, CMP, MEP, MKP (IL-7R^−^CD41^+^c-Kit^+^ Lin^−^) and CLP in donor-derived BM of G3*Terc*^*−/−*^ recipient mice treated with or without 3TC (*n* ≥ 6). **t** Absolute number of LT, ST, MPP, GMP, CMP, MEP, MKP, and CLP in recipient-derived BM of G3*Terc*^*−/−*^ recipient mice treated with or without 3TC (*n* ≥ 7). *p < 0.05; **p < 0.01; ***p < 0.001; ****p < 0.0001

To further illustrate the critical function of L1 in regulating cGAS signaling and HSC function, we administered the L1 reverse transcription inhibitor 3TC to G3*Terc*^*−/−*^ mice (Supplementary Fig. S3a). We found that the cytosolic accumulation of L1 cDNA (Fig. 1f, Supplementary Fig. S3b), the expression of 2’3’-cGAMP (Fig. 1g), and the phosphorylation levels of TBK1, IRF3, and NF-κB p65 were significantly decreased in G3*Terc*^*−/−*^ mice treated with 3TC (Supplementary Fig. S3c), but the cytosolic accumulation of L1 cDNA and the cGAS signaling was not affected in WT mice treated with 3TC (Fig. 1f–g, Supplementary Fig. S3b, d). Consequently, the cytokines production were dramatically reduced in BM cells and plasma of G3*Terc*^*−/−*^ mice treated with 3TC, but not in that of WT mice treated with 3TC(Fig. 1h, Supplementary Fig. S3e, f). Furthermore, Depletion of L1 significantly decreased cytokines expression in BM cells of G3*Terc*^*−/−*^ mice (Fig. 1i). To verify whether reduced inflammation by suppression of L1 could rescue the impaired HSC maintenance and function in telomere dysfunctional mice as we previous reported,^1^ we performed flow cytometry analysis and the results showed that 3TC treatment could recover the frequency of long-term HSCs (LTs) and multipotential progenitors (MPPs) in telomere dysfunctional mice, and 3TC treatment had no effect on the proportion of HSCs in WT mice (Fig. 1j, Supplementary Fig. S4a). Notably, competitive transplantation experiment revealed a significant improvement in the repopulating capacity of HSCs isolated from G3*Terc*^*−/−*^ mice treated with 3TC, but without affected the lineage distribution at steady state (Fig. 1k–l, Supplementary Fig. S4b). Together, these results demonstrate that suppression of L1 alleviates inflammation, thereby improves the HSC maintenance and function.

In human, chronic myelomonocytic leukemia (CMML) is a chronic myeloid neoplasm of the elderly with a poor prognosis. Mice harboring an oncogenic G12D mutation in *Nras* locus (*Nras*^*G12D*^ mice) can develop a CMML-like disease. Recent study reported that inflammatory cytokines are elevated in CMML patients.^4^ To determine whether the telomere dysfunction induced inflammatory environment affects the prognosis of leukemia, we established a mouse BM transplantation model by using the BM from *Nras*^*G12D*^ mice as donor, and half lethal dose irradiated G3*Terc*^*−/−*^ or WT mice as recipients (Supplementary Fig. S5a). We found that both G3*Terc*^*−/−*^ and WT mice transplanted with *Nras*^*G12D*^ BM (G3*Terc*^*−/−*^ recipient mice and WT recipient mice) could develop a CMML-like disease, and observed that diseased mice showed enhanced hepatosplenomegaly compared to control mice (Supplementary Fig. S5b, c), and gradually increased frequency of myeloid cells in peripheral blood (PB) from both WT recipient mice and G3*Terc*^*−/−*^ recipient mice (Supplementary Fig. S5d). Notably, the G3*Terc*^*−/−*^ recipient mice showed significantly reduced survival compared with WT recipient mice (Fig. 1m). The white blood cells count and the frequency of neutrophils were significantly increased, whereas the red blood cells count, platelets count, and frequency of lymphocytes were decreased in G3*Terc*^*−/−*^ recipient mice (Supplementary Fig. S5e). The BM analysis showed that absolute number of T and B lymphocytes were significantly decreased in G3*Terc*^*−/−*^ recipient mice (Fig. 1n). The absolute number and frequency of recipient-derived LTs, megakaryocytic/ erythroid progenitors (MEPs) and common lymphoid progenitors (CLPs) were decreased in G3*Terc*^*−/−*^ recipient mice, and the absolute number and frequency of donor-derived common myeloid progenitors (CMPs), MEPs and CLPs were decreased in G3*Terc*^*−/−*^ recipient mice as well (Fig. 1o, p, Supplementary Fig. S5f–h). In addition, we found that the cytokines production were dramatically upregulated in plasma of G3*Terc*^*−/−*^ recipient mice, although IL-6 was also increased in donor mice (Supplementary Fig. S5i). Together, these results indicate that the repression of the normal hematopoiesis and increased inflammation contributes to the decreased survival of G3*Terc*^*−/−*^ recipient mice.

To determine whether improvement of HSC by L1 inhibition is beneficial in prognosis of CMML, we administered 3TC to G3*Terc*^*−/−*^ recipient mice and WT recipient mice (Supplementary Fig. S6a), and found that the 3TC treatment extended the survival of G3*Terc*^*−/−*^ recipient mice, but not WT recipient mice (Fig. 1q). The expression of cytokines in plasma of G3*Terc*^*−/−*^ recipient mice treated with 3TC were significantly decreased (Supplementary Fig. S6b). Interestingly, we found that 3TC could not relieve the hepatosplenomegaly, high proportion of myeloid cells in PB and high absolute number of myeloid cells in BM from G3*Terc*^*−/−*^ recipient mice (Fig. 1r, Supplementary Fig. S6c-e), and the absolute number and frequency of donor-derived hematopoietic stem/progenitor cells (HSPCs) were comparable between 3TC treated and untreated G3*Terc*^*−/−*^ recipient mice (Fig. 1s, Supplementary Fig. S6f). Notably, the absolute number and frequency of recipient-derived LTs, MEPs and megakaryocyte progenitors (MKPs) were dramatically increased, and the PLTs count was rescued to normal level in G3*Terc*^*−/−*^ recipient mice with 3TC treatment (Fig. 1t, Supplementary Fig. S6g, h). To explore whether 3TC treatment also contributes to the repression of donor cell (*Nras*^*G12D*^), we checked the mRNA expression and CpG methylation level of L1 in diseased mice. The results showed decreased CpG methylation at L1 promoter region in endogenous G3*Terc*^*−/−*^ BM cells, but not in endogenous WT BM cells, the donor-derived (*Nras*^*G12D*^) BM cells in WT and G3*Terc*^*−/−*^ recipient mice (Supplementary Fig. S7a). The expression level of L1 in different groups were consistent with the CpG methylation levels (Supplementary Fig. S7b). Altogether, these results indicate that the suppression of L1 by 3TC treatment in CMML mice reduces telomere dysfunction induced inflammation and attenuates the impaired hematopoiesis, thereby extended survival of the diseased mice, probably due to the improvement of HSPCs maintenance.

In summary, we found that L1 activation is responsible for the cGAS signaling induced inflammatory responses in G3*Terc*^*−/−*^ mice. 3TC treatment attenuated the aging-associated decline of HSC maintenance and function, thereby extended the survival of G3*Terc*^*−/−*^ recipient mice transplanted with oncogenic *Nras*^*G12D*^ BM. Our findings suggest that reverse transcriptase inhibition may serve as a new therapeutic strategy for patients suffering from age-related disorders.

## Supplementary information

Supplementary information
