# Supplementary material for: L1 drives HSC aging and affects prognosis of chronic myelomonocytic leukemia
Source: Signal Transduct Target Ther. 2020 Sep 19;5:205. doi: 10.1038/s41392-020-00279-4 (PMC7502075; doi:10.1038/s41392-020-00279-4)
Supplement: Supplementary file 1 — Supplementary information [file 41392_2020_279_MOESM1_ESM.docx]

**Supplemental Material for**

**L1 drives HSC aging and affects prognosis of chronic myelomonocytic leukemia**

Ying Wang^1, §, *^, Jin-ping Zheng^2,§^, Ying Luo^3^, Junyi Wang^1^, Lingjie Xu^1^, Jinyong Wang^4^, John M. Sedivy^5^, Zhangfa Song^6, *^, Hu Wang^1, 3, *^, Zhenyu Ju^1, 3, *^

^§^Y.W. and JP.Z. contributed equally to this work.

^*^To whom correspondence may be addressed. Email: zhenyuju@163.com, wanghu19860315@163.com, songzhangfa@zju.edu.cn, flashingdancer@163.com

**This file includes:**

Materials and Methods

References

Figures. S1 to S7

**Materials and methods**

**Mice**

G3*Terc^-/-^* mice were used as donor or recipient for HSCs/bone marrow transplantation. Mice bearing the conditional oncogenic *Nras* (Lox-stop-Lox *Nras*^G12D^) mutation were gifted from Professor Jinyong Wang[^1^](#_ENREF_1), and were crossed to Vav-Cre mice to generate mice carrying both alleles (LSL *Nras*^G12D/+^; Vav-Cre), which served as donor mice were used in CMML model. *cGAS^-/-^* mice purchased from Institut Clinique de la Souris (ICS) were crossed to *Terc^-/-^* mice to generate G3*Terc^-/-^cGAS^-/-^* mice. For transplantation, all recipient mice are age- and sex-matched 8 to 10-week-old mice. All experiments were approved by the Animal Care and Ethics Committee at Hangzhou Normal University. G3*Terc^-/-^* mice were administered 2 mg/mL 3TC in drinking water for 1 month. For the CMML model, the recipient mice were administered 2 mg/mL 3TC in drinking water for 6 month after transplantation.

**Flow cytometry**

BM cells were isolated by crushing the bones from femurs, tibiae, iliac crests and spine in PBS, and filtered through a sterile nylon mesh. Peripheral blood was collected by puncture of the retro-orbital plexus. Then the cells were counted and stained with antibody cocktail prepared in PBS buffer for at least 30min at 4℃. After all the staining steps, red blood cells were lysed with 1 × lysis buffer (BD), washed in PBS, and centrifuged at 1400 rpm for 5min at 4℃. The pellet was re-suspended in staining buffer for analysis. For sorting of specific populations, cells were sorted directly into PBS contain 0.2% BSA for the following experiments.Prepared samples were analyzed by LSR Fortessa cell analyzer or sorted on an Influx cell sorter (BD Biosciences). Data were analyzed using FACSDiva (BD) and FlowJo 9.9.3 (TreeStar, Ashland, OR, USA).

**Reconstitution assay**

Competitive reconstitution was performed by using the congenic CD45.1/CD45.2 mouse system. 500 LT cells from donor mice mixed with 5×10^5^ competitor BM cells or 1×10^6^ BM were transplanted into lethally (9Gy) / half lethally (4.5Gy) irradiated recipient mice, and BM analysis was performed when the mice were sacrificed. Recipient mice were given antibiotics via drinking water for two weeks after injection. PB were collected from the recipient mice and analyzed for chimerism and lineage distribution every 4 weeks.

**Luminex assay**

Total 50ul plasma of each mice was used for cytokines detection with Mouse Premixed Multi-Analyte Kit (R&D Systems, Minneapolis, MN), according to the manufacturer’s instructions. The data were collected from the Luminex 200 (Luminex Corporation, Austin, TX).

**2’3’-cGAMP Analysis by HPLC-Electrospray Ionization Tandem Mass Spectrometry**

2’3’-cGAMP analysis was performed as previously described[^2^](#_ENREF_2). 2 × 10^7^ bone marrow cells were extracted using cold 80% MeOH and 2% acetic acid and placed at -80 °C for 1 h. The extract was dried down in a SpeedVac (Thermo Fisher Scientific) and overlaid with 500μl of cold methanol/water (50/50, v/v). After rigorous vortexing, the debris was pelleted by centrifugation at 5000 × g and 4°C for 5 min. The supernatant was transferred to a new tube and was then transferred to a high-performance liquid chromatography (HPLC) vial for the metabolomics study. 2’ 3’-cGAMP analyses was performed using a liquid chromatography-tandem mass spectrometry (LC-MS/MS) approach. Separation was achieved on a Agilent ZORBAX Bonus-RP HPLC column (5um, 2.1 x 150mm) using a Agilent Technologies 1260 Infinity HPLC system. The mobile phases employed were acetonitrile (A) and 10mM ammonium acetate in water (B).

LC-MS analyses were performed in negative electrospray mode on a AB SCIEX QTRAP 4500 with online separation and with a Agilent Technologies 1260 Infinity HPLC system. The conditions used for 2’3’-cGAMP analyses were: column, ZORBAX Bonus-RP 5um, 2.1 x 150mm (Agilent); mobile phase A, acetonitrile; mobile phase B, 10mM ammonium acetate in water; The gradient program was as follows: 0–0.5 min, 5%-100% A; 0.5–3 min, 100%A; 3-3.1 min, 100% A-5%A; 3.1-13min, 5% A. The column and the sample was at room temperature 22℃. The flow rate was 0.3 ml/min, and injection volume was 10 μl. The mass spectrometer was an QTRAP 4500 (Applied Biosystems SCIEX) with electrospray ionization (ESI) source in multiple reaction monitoring (MRM) mode. Sample analysis was performed in negative mode. Declustering potential (DP) is -135V（negative. The MRM MS/MS detector conditions were set as follows: curtain gas 30 psi; ion spray voltages, -4500 V (negative); temperature, 500°C; ion source gas 1, 55 psi; ion source gas 2, 55 psi. The qualitative ion pair is 673/344（m/z, CE -45V. The qualitative ion pair is 673/328（m/z）, CE -45V and 673/211, CE -51V. Cell samples were analyzed in a randomized order, and MRM data were acquired using Analyst 1.6.1 software (Applied Biosystems SCIEX).

**DNA methylation assay**

L1 promoter DNA methylation analysis was performed by Generay Biotech Co., Ltd (Shanghai, China) following the previously published protocol. Briefly, around 10 clones were sequenced from BM cells of WT, G3*Terc^-/-^*, WT recipient and G3*Terc^-/-^* recipient mice. The bisulfite genomic sequencing primers for L1 promoter where 12 CpGs used for the methylation analysis are as follows[^3^](#_ENREF_3). p1: 5’-3’ GTTGAGGTAGTATTTGTGTGGGT and p2: 5’-3’ TTCCAAAAACTATCAA ATTCTCTAACA, BiQ Analyzer was used to analyze the data collected in sequencing.

**Detection of cDNA content of L1 in cytosolic extracts**

2×10^7^ bone marrow cells were each divided into two aliquots of equal volume. One aliquot was using extract total DNA, which served as normalization controls for total DNA. The second aliquot was resuspended in 500μL buffer containing 150mM NaCl, 50mM Hepes (pH 7.4), and 25μg/mL digitonin. The homogenates were incubated end-over-end for 4 min to allow for selective plasma membrane permeabilization and then centrifuged three times at 980 × g for 5 min to pellet intact cells. The cytosolic supernatants were transferred to fresh tubes and spun at 17,000 × g for 25 min to pellet any remaining cellular debris, yielding cytosolic preparations free of nuclear, mitochondrial, and endoplasmic reticulum contamination. DNA was then isolated from these pure cytosolic fractions using QIAquick Nucleotide Removal Columns (Qiagen). Quantitative real-time PCR was performed on both whole-cell extracts and cytosolic fractions using nuclear DNA primers (*Tert*) and L1 primers and the cycle threshold (CT) values obtained for L1 abundance for whole-cell extracts served as normalization controls for the L1 values obtained from the cytosolic fractions. This allowed for effective standardization among samples and controlled for any variations in the total amount of L1 in samples. Using this digitonin method, no nuclear *Tert* DNA was detected in the cytosolic fractions, indicating nuclear lysis did not occur.

**Antibodies**

The antibodies used in flow cytometry analysis included CD4 (RM4-5, 1:100), CD8 (53-6.7, 1:100), CD11b (M1/70, 1:150), Gr-1 (RB6-8C5, 1:150), Ter-119 (TER-119, 1:100), B220 (RA3-6B2, 1:100), CD34 (RAM34, 1:100), CD45.1 (A20, 1:100), CD45.2 (104, 1:100), Flt3 (A2F10, 1:100), Sca1 (E13-161.7, 1:100), c-Kit (ACK2, 1:100), CD16/32 (93, 1:100), CD41 (WMReg30), IL-7R (A7R34), and streptavidin， which all from BD/ebioscience/Biolegend. Antibodies used in western blotting included p-TBK1 (5483, CST), TBK1 (3504, CST), p-IRF3 (29047, CST), IRF3 (4302, CST), p-NF-κB p65 (3033, CST), NF-κB p65 (8482, CST), ORF1p (ab216324, Abcam), and Tubulin (ab52866, Abcam).

**Quantitative real-time PCR**

For each mouse, RNA from BM were isolated using Trizol（Invitrogen）and were reversed into cDNA using PrimeScript^TM^ RT reagent kit with gDNA Eraser (Takara). All PCR were carried in triplicate for each cDNA sample in the ABI PRISM 7300 Sequence Detection System (Applied Biosystems) and the following primers (5’-3’): L1, ATGGCGAAAGGCAAACGTAAG (F) and ATTTTCGGTTGTGTTGGGGTG (R); IFNα, GCCTGTTCTCTAGGATGTGAC (F) and CCTTCCTGTCCTTCAGG CAGG (R); IFNβ, CAGCTCCAAGAAAGGACGAAC (F) and GGCAGTGTAA CTCTTCTGCAT (R); IL-6, CCAAGAGGTGAGTGCTTCCC (F) and CTGTTGT TCAGACTCTCTCCCT (R); IL-17A, TTTAACTCCCTTGGCGCAAAA (F) and CTTTCCCTCCGCATTGACAC (R); TNFα, CCCT CACACTCAGATCATCTTCT (F) and GCTACGACGTGGGCTACAG (R); CXCL1, CTGGGATTCACCTCA AGAACATC (F) and CAGGGTCAAGGCAAGCCTC (R); CXCL10, CCAAG TGCTGCCGTCATTTTC (F) and GGCTCGCAGGGATGATTTCAA (R).

**RNA interference**

BM cells were transfected with 60 nM mixed siRNAs specific for mouse L1 using Lipofectamine^TM^ 2000 transfection reagent (Invitrogen). After 72 h of transfection, cells were harvested for quantitative real-time PCR analysis. Two L1 siRNAs were synthesized (Shanghai GenePharma, Co., Ltd.) as previously reported[^4^](#_ENREF_4). The sequence (5’-3’) for L1-siRNA1 was CAACAAAGATAGAGAACTTTT; and the sequence for L1‑siRNA2 was GGGCATATATCCAGAAGAATT.

**Statistics**

We carried out statistical analyses using GraphPad Prism software. Two-way ANOVA was used to generate P-values for data of chimerism analysis in PB. The unpaired Student’s t-test was used to generate P-values for all other datasets. p<0.05 was considered significant. * Represents p<0.05, ** represents p<0.01, *** represents p<0.001, and **** represents p<0.0001. The reported values are means ± SEM, Sample size (n) represents biological replicates, noted in the figure legends.

**References**

1. Wang, J. *et al.* Endogenous oncogenic Nras mutation promotes aberrant GM-CSF signaling in granulocytic/monocytic precursors in a murine model of chronic myelomonocytic leukemia. *Blood* **116**, 5991-6002 (2010).

2. Bai, J. *et al.* DsbA-L prevents obesity-induced inflammation and insulin resistance by suppressing the mtDNA release-activated cGAS-cGAMP-STING pathway. *Proceedings of the National Academy of Sciences of the United States of America* **114**, 12196-12201 (2017).

3. Bedrosian, T.A., Quayle, C., Novaresi, N. & Gage, F.H. Early life experience drives structural variation of neural genomes in mice. *Science* **359**, 1395-1399 (2018).

4. Percharde, M. *et al.* A LINE1-Nucleolin Partnership Regulates Early Development and ESC Identity. *Cell* **174**, 391-405 e319 (2018).

**
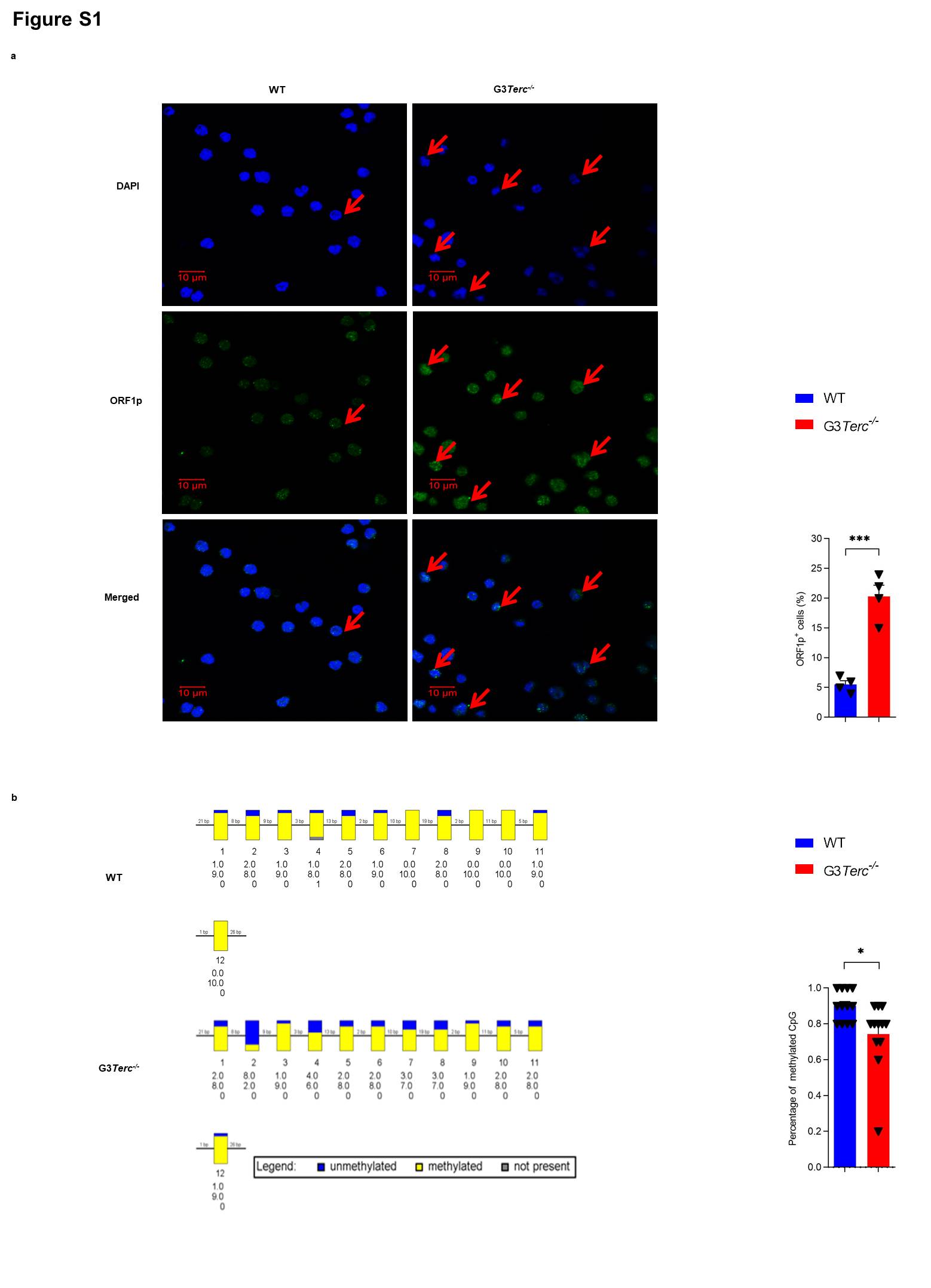
**

**Figure S1 L1 is activated in BM cells of Telomere dysfunctional mice.** (a) BM cells of WT and G3*Terc^-/-^* mice were immunostained with ORF1p and quantification as indicated (green) and DAPI (blue) (n=4). (b) Quantification of CpG island methylation status of promoter regions of L1 on chromosomes. Yellow and blue bars denote the frequencies of methylated and unmethylated CpG islands respectively, at each position (n=12). *p < 0.05; **p < 0.01; ***p < 0.001; ****p < 0.0001.


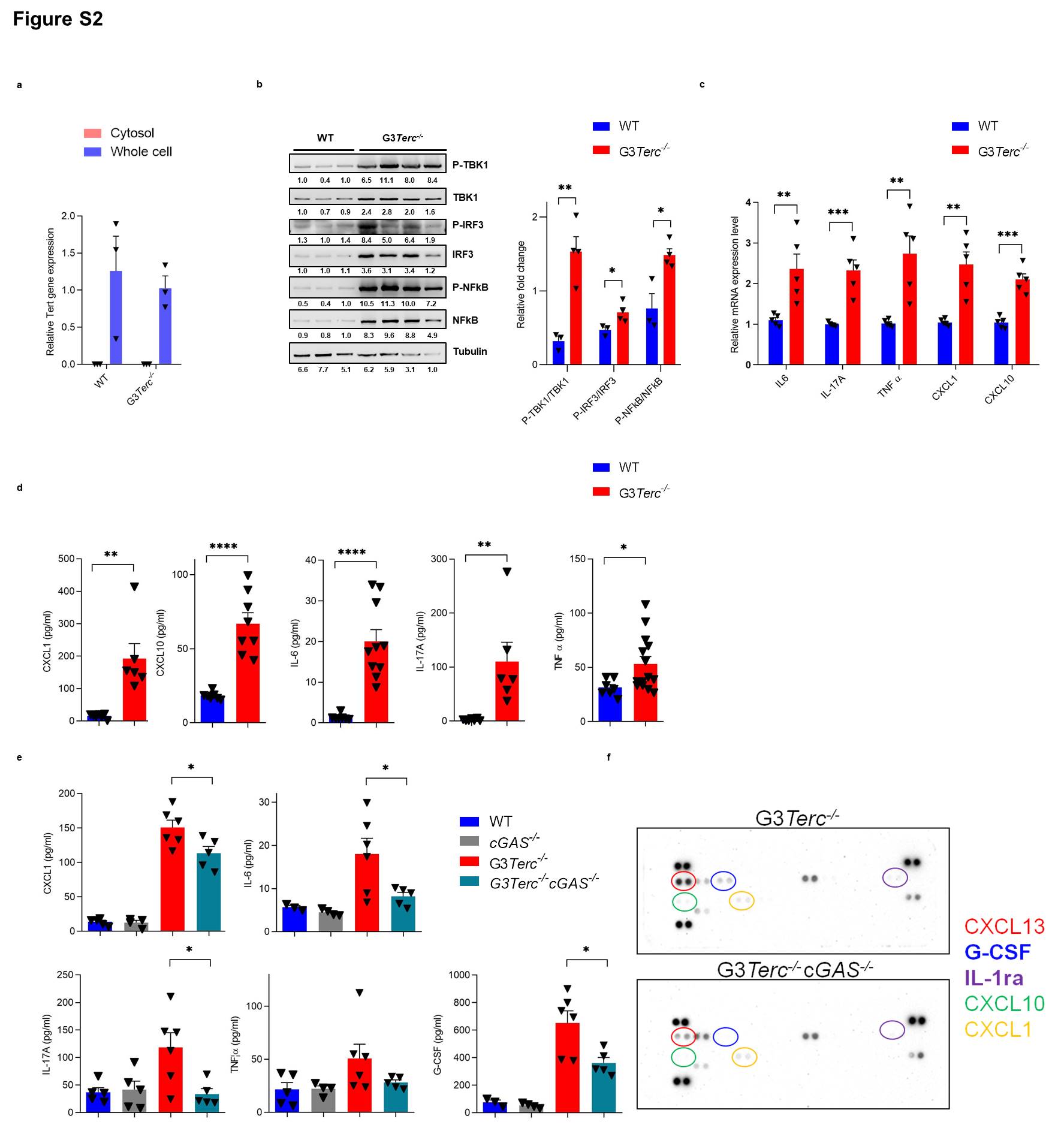


**Figure S2 cGAS signaling is responsible for L1 induced inflammation in telomere dysfunctional mice.** (a) The purity of the cytosolic fraction was confirmed by nuclear markers Tert DNA detection. Nuclear encoded Tert gene expression was quantitated via qPCR in whole cell and cytosolic extracts from freshly purified BM cells of WT and G3*Terc^-/-^* mice (n=3). (b) The protein levels of p-TBK1, TBK1, p-IRF3, IRF3, p-NF-κB p65, NF-κB p65 and Tubulin were determined by western blot in BM of WT and G3*Terc^-/-^* mice. The graph represents the relative fold change of the indicated proteins. (c) Q-PCR analysis of the relative mRNA expression levels of IL-6, IL-17A, TNFα, CXCL1 and CXCL10 in BM of WT and G3*Terc^-/-^* mice (n=5). (d) Murine plasma cytokines CXCL1, CXCL10, IL-6, IL-17A and TNFα were quantified using luminex cytokine assay in WT and G3*Terc^-/-^* mice (n≥6). (e) Murine plasma cytokines CXCL1, IL-6, IL-17A, TNFα, and G-CSF were quantified using luminex cytokine assay in WT, c*GAS^-/-^*, G3*Terc^-/-^* and G3*Terc^-/-^*c*GAS^-/-^* mice (n≥3). (f) Representative photographs of cytokines array using plasma from G3*Terc^-/-^* and G3*Terc^-/-^*c*GAS^-/-^* mice. Colored circles mark differentially expressed cytokines. *p < 0.05; **p < 0.01; ***p < 0.001; ****p < 0.0001.


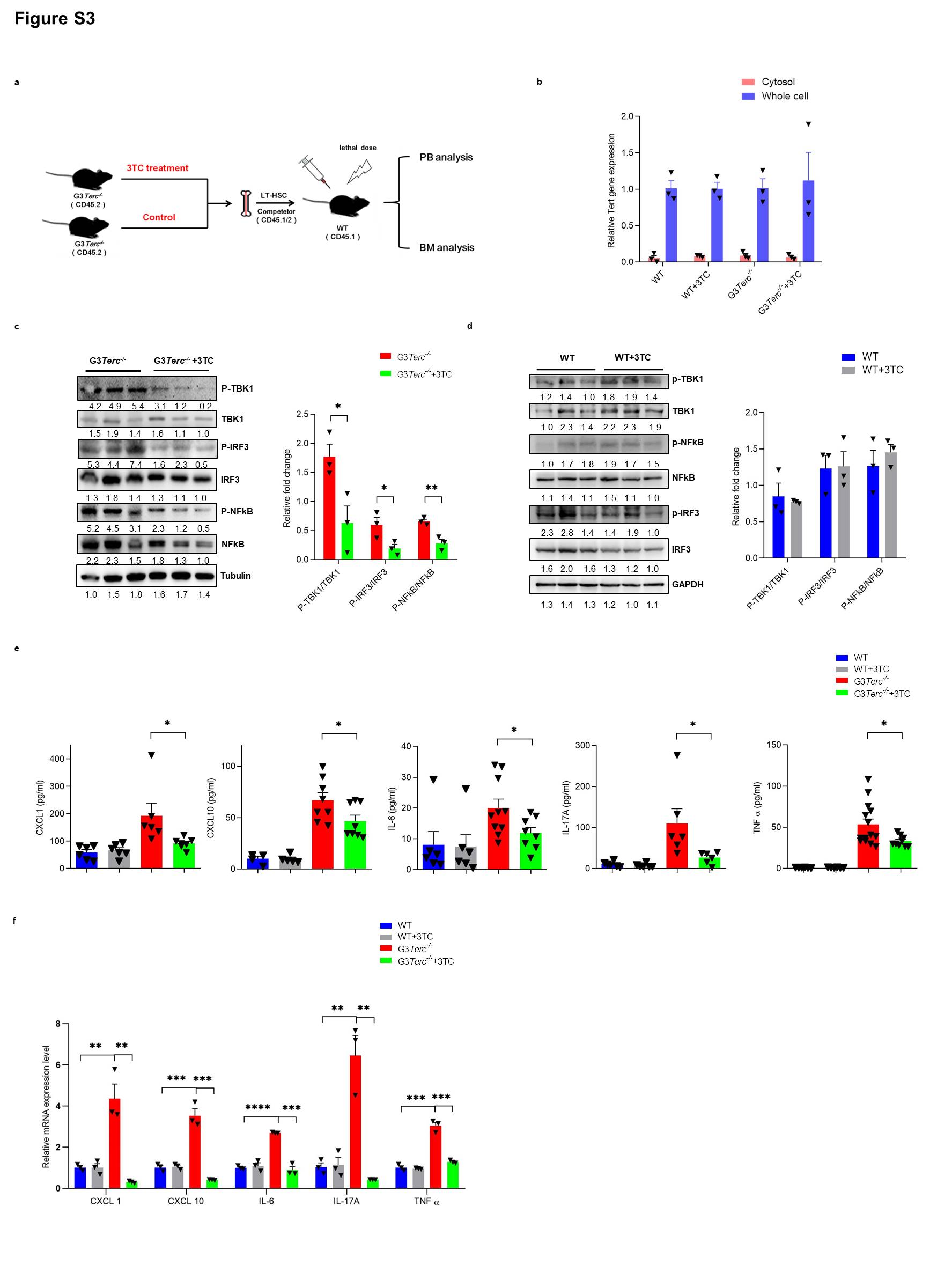


**Figure S3 Suppression of L1 alleviated cGAS signaling mediated inflammation in telomere dysfunctional mice.** (a) Experimental schematic for the reconstitution assay. (b) Nuclear encoded Tert gene expression was quantitated via qPCR in whole cell and cytosolic extracts from freshly purified BM cells of WT or G3*Terc^-/-^* mice treated with or without 3TC (n=3). (c, d) The protein levels of p-TBK1, TBK1, p-IRF3, IRF3, p-NF-κB p65, NF-κB p65 and Tubulin were determined by western blot in BM of G3*Terc^-/-^* mice (c) or WT mice (d) treated with or without 3TC. The graph represents the relative fold change of the indicated proteins. (e) Murine plasma cytokines CXCL1, CXCL10, IL-6, IL-17A and TNFα were quantified using luminex cytokine assay in WT or G3*Terc^-/-^* mice treated with or without 3TC (n≥4). (f) Q-PCR analysis of the relative mRNA expression levels of CXCL1, CXCL10, IL-6, IL-17A and TNFα in BM of WT or G3*Terc^-/-^* mice treated with or without 3TC (n=3). *p < 0.05; **p < 0.01; ***p < 0.001; ****p < 0.0001.


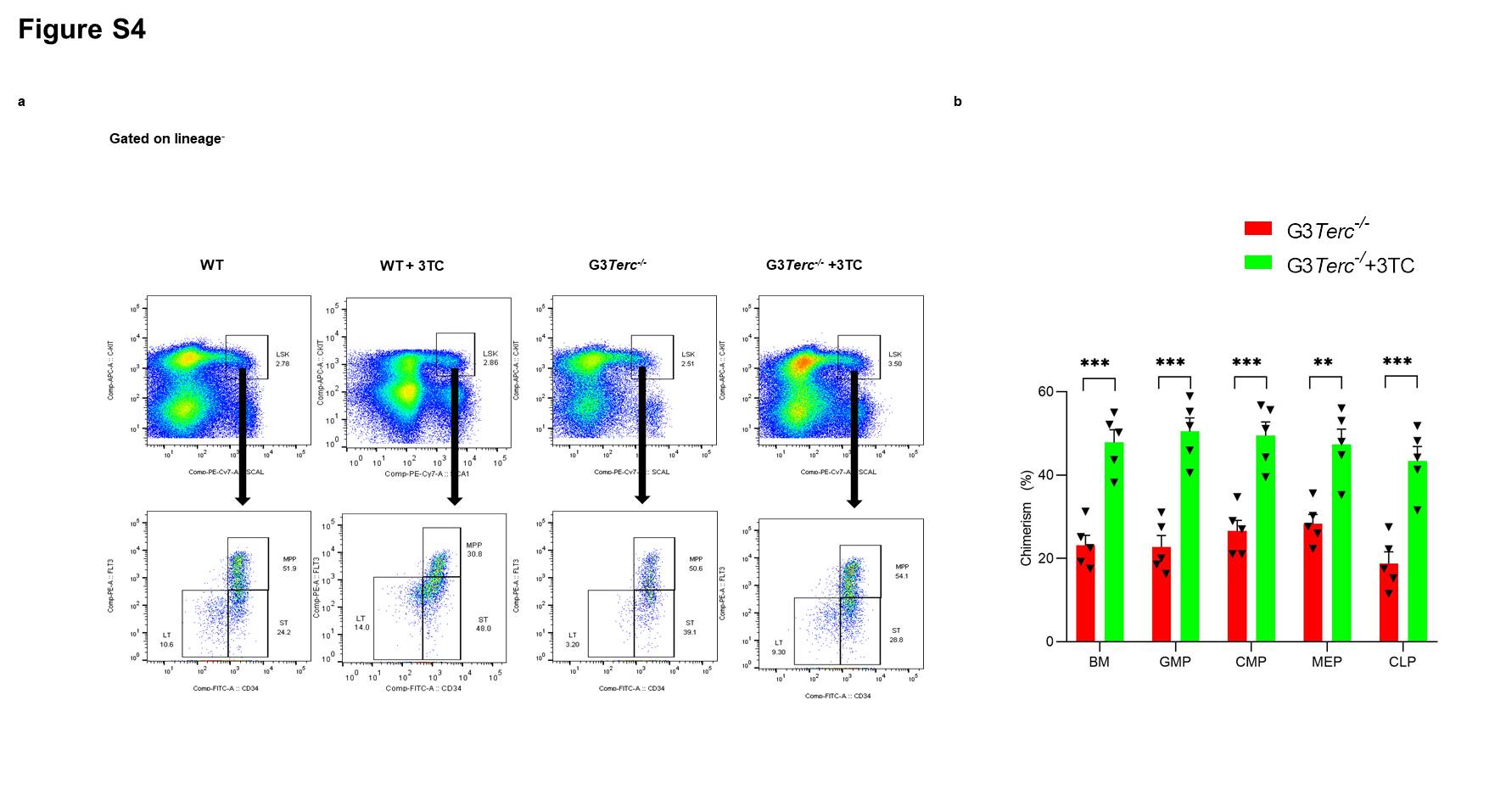


**Figure S4 Suppression of L1 rescued HSC maintenance and function in telomere dysfunctional mice.** (a) Representative FACS plot showing the gating of LT, ST, MPP in BM cells of WT or G3*Terc^-/-^* mice treated with or without 3TC. (b) Percentage of donor-derived cells in BM, GMP, CMP, MEP and CLP at 16 weeks after transplantation (n=5). *p < 0.05; **p < 0.01; ***p < 0.001; ****p < 0.0001.

**
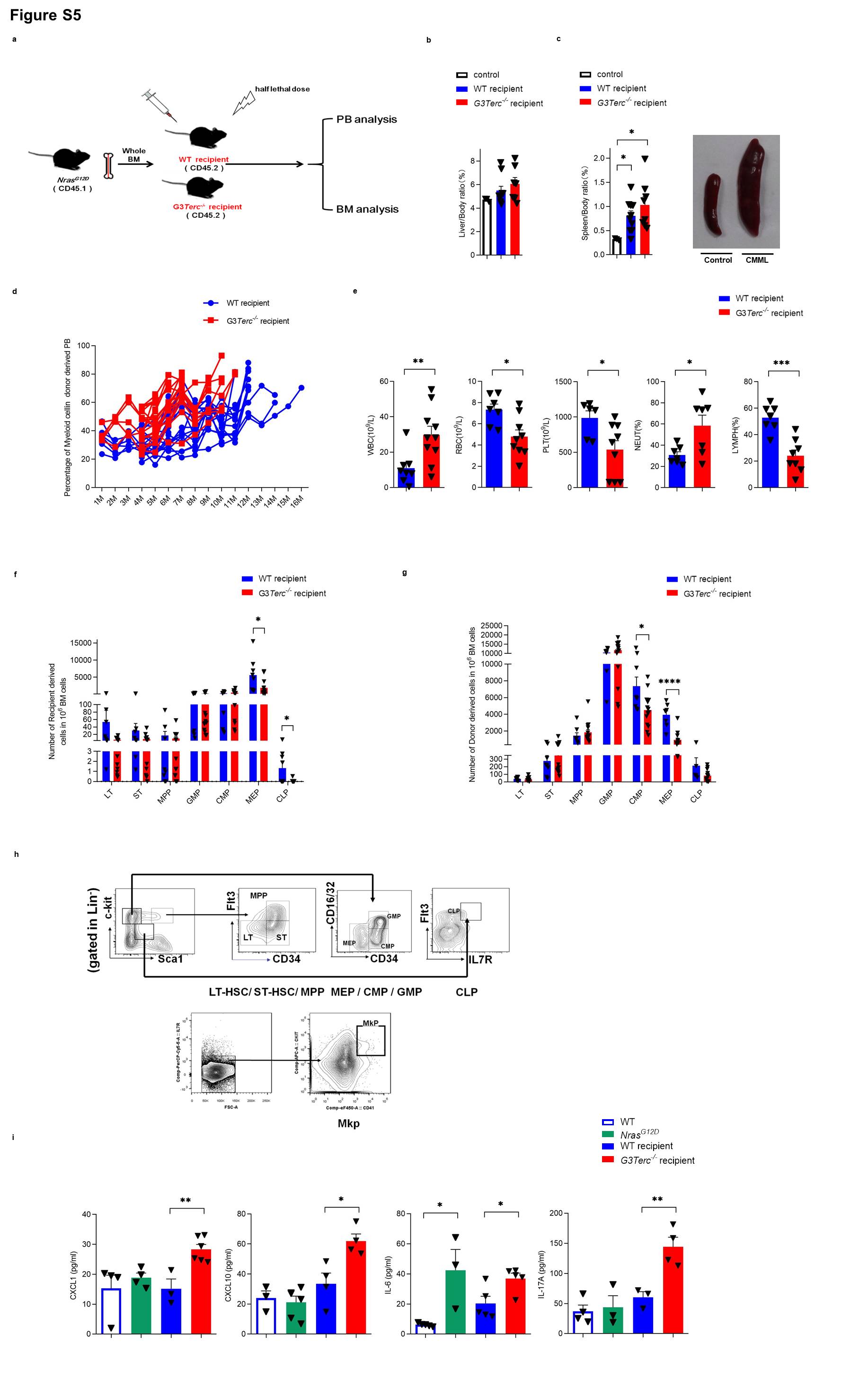
**

**Figure S5 Telomere dysfunctional inflammatory BM environment decreased the survival of CMML mice.** (a) Experimental schematic for the reconstitution assay. (b) Liver/Body weight ratio (%) in control, WT recipient and G3*Terc^-/-^* recipient mice (n≥3). (c) Spleen/Body weight ratio (%) in control, WT recipient and G3*Terc^-/-^* recipient mice (left); Splenomegaly in a representative recipient mouse that developed a CMML-like disease (right) (n≥3). (d) Dynamic percentages of donor-derived myeloid cells in PB of WT recipient and G3*Terc^-/-^* recipient mice (n≥5). (e) The PB count of white blood cells (WBC), red blood cells (RBC), platelets (PLT), and the frequency of neutrophils (NEUT) and lymphocytes (LYMPH) in WT recipient mice and G3*Terc^-/-^* recipient mice (n≥6). (f) Numbers of LT, ST, MPP, GMP, CMP, MEP, and CLP per million recipient-derived BM of WT recipient and G3*Terc^-/-^* recipient mice (n≥8). (g) Numbers of LT, ST, MPP, GMP, CMP, MEP, and CLP per million donor-derived BM of WT recipient and G3*Terc^-/-^* recipient mice (n≥5). (h) Representative FACS gating strategy for the analysis of LT-HSC,ST-HSC, MPP, GMP, CMP, MEP, CLP and MKP in BM cells. (i) Murine plasma cytokines CXCL1, CXCL10, IL-6, IL-17A were quantified using ELISA in WT, *Nras^G12D^*, WT recipient and G3*Terc^-/-^* recipient mice (n≥3). *p < 0.05; **p < 0.01; ***p < 0.001; ****p < 0.0001.

**
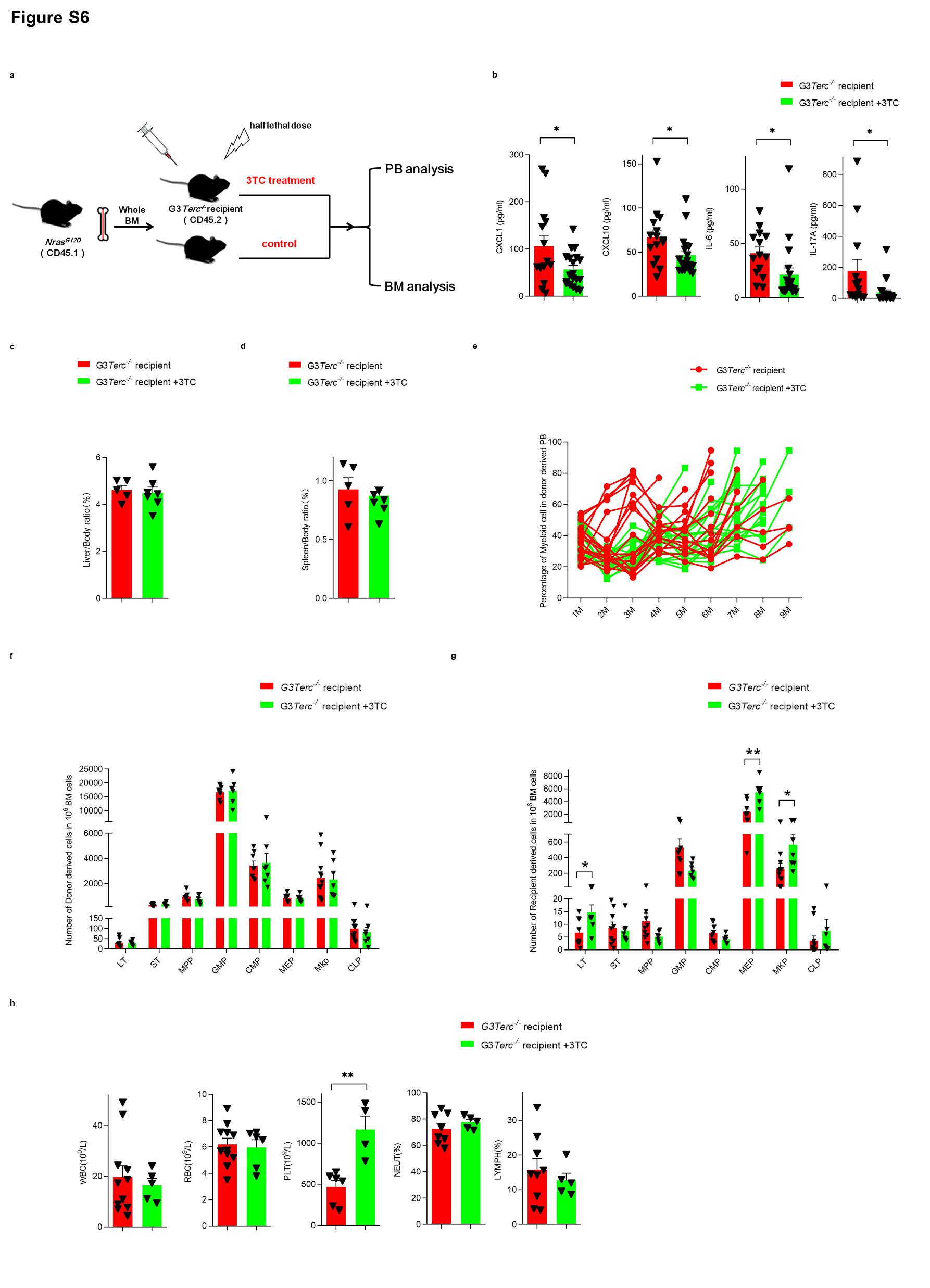
**

**Figure S6 3TC treatment increased the survival of CMML mice through improved hematopoiesis.** (a) Experimental schematic for the reconstitution assay. (b) Murine plasma cytokines CXCL1, CXCL10, IL-6 and IL-17A, were quantified using luminex cytokine assay in G3*Terc^-/-^* recipient mice treated with or without 3TC (n≥13). (c) Liver/Body weight ratio (%) in G3*Terc^-/-^* recipient mice treated with or without 3TC (n≥5). (d) Spleen/Body weight ratio (%) in G3*Terc^-/-^* recipient mice treated with or without 3TC (n≥5). (e) Dynamic percentages of donor-derived myeloid cells in PB of G3*Terc^-/-^* recipient mice treated with or without 3TC (n≥19). (f) Numbers of LT, ST, MPP, GMP, CMP, MEP, MKP and CLP per million donor-derived BM of G3*Terc^-/-^* recipient mice treated with or without 3TC (n≥7). (g) Numbers of LT, ST, MPP, GMP, CMP, MEP, MKP and CLP per million recipient-derived BM of G3*Terc^-/-^* recipient mice treated with or without 3TC (n≥7). (h) The PB count of WBC, RBC, PLT, and the frequency of NEUT and LYMPH in G3*Terc^-/-^* recipient mice treated with or without 3TC (n≥4). *p < 0.05; **p < 0.01; ***p < 0.001; ****p < 0.0001.


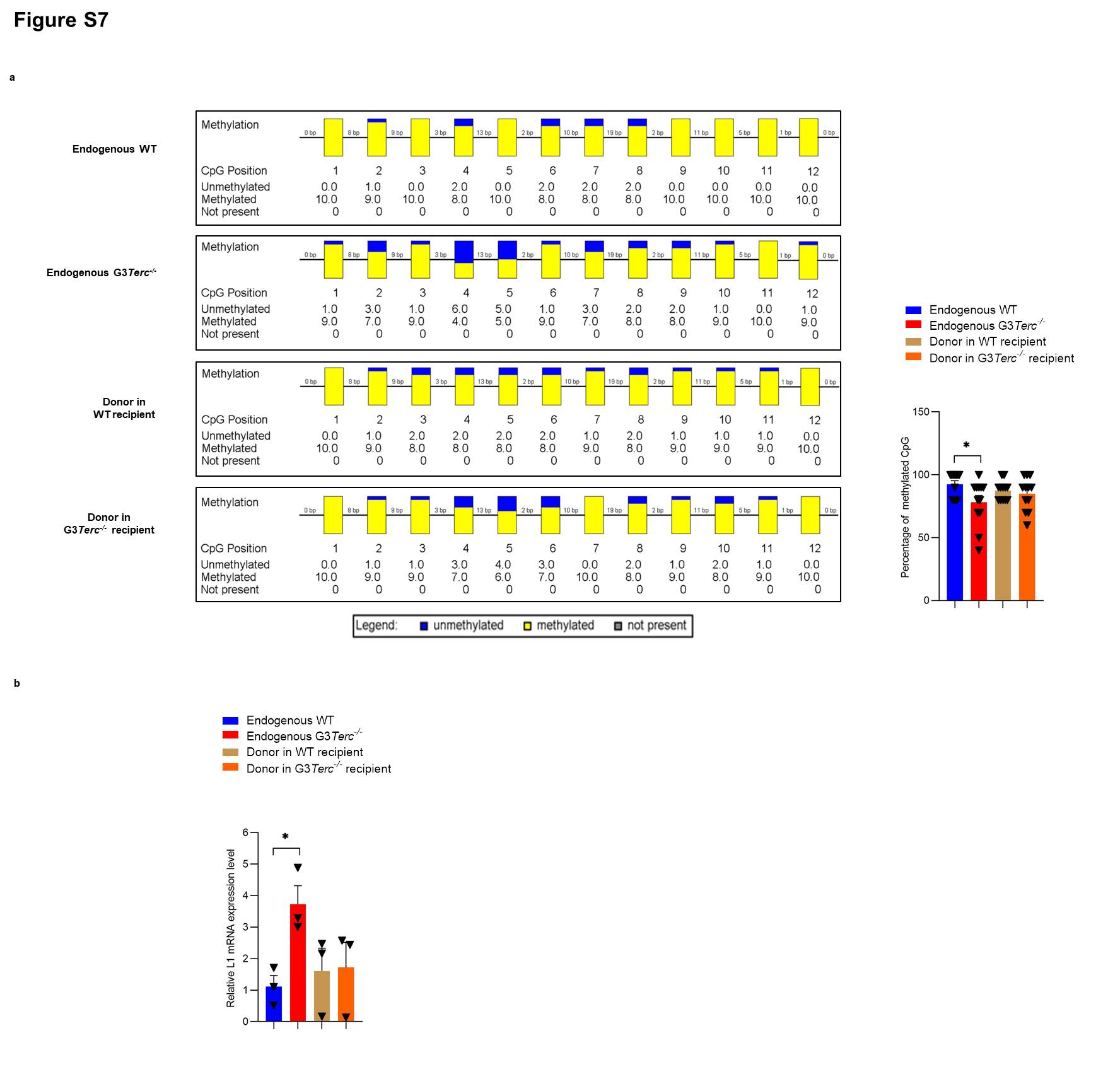


**Figure S7** **The methylation state and L1 expression level in CMML disease mice.** (a) The endogenous WT or G3*Terc^-/-^* BM cells (CD45.2) were separated from the donor-derived (*Nras^G12D^*, CD45.1) BM cells in transplanted mice by flow cytometry. Quantification of CpG island methylation status of promoter regions of L1 on chromosomes in BM cells of Endogenous WT，Endogenous G3*Terc^-/-^*，Donor in WT recipient and Donor in G3*Terc^-/-^* mice. Yellow and blue bars denote the frequencies of methylated and unmethylated CpG islands respectively, at each position (n=12). (b) Q-PCR analysis of the relative mRNA expression levels of L1 in BM cells of Endogenous WT, Endogenous G3*Terc^-/-^*, Donor in WT recipient and Donor in G3*Terc^-/-^* mice (n=3).
